# An Unusual and Rare Presentation of Diffuse Tophaceous Gout

**DOI:** 10.7759/cureus.30121

**Published:** 2022-10-10

**Authors:** Rebecca A Levy-Bedoya, Daniela Pi Noa, Aashish Dewan, Sergio Tierrablanca, Neil H Strauss

**Affiliations:** 1 Family Medicine, Broward Health Medical Center, Fort Lauderdale, USA; 2 College of Medicine, Nova Southeastern University Dr. Kiran C. Patel College of Osteopathic Medicine, Davie, USA; 3 Advanced Foot, Ankle, and Wound Care, Broward Health Medical Center, Fort Lauderdale, USA

**Keywords:** gout, abscess, polyarticular arthritis, tophi, tenosynovitis

## Abstract

Gout is a chronic disease characterized by recurrent attacks on joints from monosodium urate crystal deposition causing inflammation and severe pain. Patients at increased risk of developing gout include those with obesity, high consumption of alcohol or high-purine foods, genetic causes, and medication side effects. Typically, there are three stages of disease progression: acute, inter-critical, and chronic. Monoarticular joint disease is common however polyarticular gouty arthritis can result after years of acute flares. The chronic nature of the disease forms tophi, which are generally painless solid urate crystal collections. We present an unusual case of a 33-year-old male whose initial presentation was severe tophaceous gout affecting multiple joints, including bilateral elbows, knees, as well as hand and foot joints. His presentation was unique in that the tophi were not firm as expected, but were erythematous, tender, and fluctuant resembling an abscess. Laboratory and imaging studies confirmed the diagnosis of tophaceous gout and the patient’s symptoms improved after starting systemic steroid therapy and colchicine. A multidisciplinary effort involving the medicine team and infectious disease, podiatry, and rheumatology consultants was essential in reaching the diagnosis. This case highlights the importance of keeping a broad differential diagnosis in a patient with polyarticular lesions and considering gout even with an atypical presentation such as in our patient.

## Introduction

Gout is a debilitating disease, characterized by the deposition of monosodium urate (MSU) crystals into joints causing joint inflammation and severe pain. Excessive uric acid production through purine metabolism or impaired urate clearance through the kidney and gastrointestinal tract, leading to hyperuricemia, can eventually advance to gout. Causes of hyperuricemia can be due to a diet rich in purines, excessive alcohol consumption, genetic causes, medication side effects, and obesity. High purine loads as the etiology of hyperuricemia are related to the intake of seafood and red [1]. Additionally, patients with psoriasis have been found to have increased serum levels of uric acid [2]. Commonly known drugs leading to hyperuricemia include cytotoxic chemotherapy, diuretics, and anti-tubercular drugs. Loop diuretics and thiazide diuretics are some of the more well-known medications to cause gout, where increased levels of serum uric acid can be noted within just a few days of starting treatment [3]. It is also worth noting the genetic causes of gout. For example, there are two known uric acid transporters, called SLC2A9 and ABCG2, where alterations in these genes can cause hyperuricemia [4]. Additionally, deficiency of the enzyme hypoxanthine-guanine phosphoribosyl transferase, such as in Lesch-Nyhan disease, results in the overproduction of urate which can lead to acute or chronic kidney disease [5].

In the United States, the prevalence of gout in the adult population is about 3.9%, which is nearly 9.2 million people. Gout is most commonly seen in middle-aged and older adults, with a higher risk in patients with comorbidities such as cardiovascular disease, diabetes, chronic kidney disease, and obesity [6]. Normal serum urate levels in males peaks at puberty and range between 5-6 mg/dL, whereas normal urate levels in females average 1-1.5 mg/dL [7-11].

Classically, three clinical stages that tend to occur sequentially have been identified in gout patients: acute gout flare, inter-critical phases, and chronic gouty arthritis or diffused tophaceous gout. Typically, gout flares are monoarticular at the time of presentation and can be initiated by proinflammatory triggers such as specific diets, trauma, surgery, or certain drugs [11,12]. Clinically, the patient will demonstrate severe pain, swelling, erythema, and warmth of the affected joint. Symptoms peak between 12-24 hours after onset and can last a few days to weeks. Most gouty flares resolve within weeks, whether the patient seeks treatment or not [13]. Polyarticular gouty flares account for less than 20% of cases at their initial presentation, however, are more common later in the disease course. Polyarticular gout flares are more prevalent in the hospitalized population and can mirror sepsis in some cases. Generally, polyarticular flares develop in a migratory fashion, affecting the lower extremity joints initially and traveling to the upper extremity joints and bursa [9,13].

After resolution of the initial gouty flare, patients enter an inter-critical phase which is generally asymptomatic and varies in duration depending on individual characteristics and whether the treatment has been initiated. Patients who present with higher serum urate levels at baseline tend to experience flares more frequently. Likewise, those who defer treatment usually develop a second flare within two years due to the persistently elevated serum uric acid levels. Moreover, shorter asymptomatic periods have been observed later in the disease course [14]. Acute gout is a distressing and crippling state which can lead to joint erosion if left untreated. Chronic gout can lead to tophi, which are painless solid urate crystal collections encompassing multiple joints and is associated with chronic inflammation and destructive changes in the surrounding connective tissue [1]. Less commonly, tophaceous gout presents in patients with no previous history of gouty flares [12,13].

Diagnosing and treatment of gout early in the disease process is crucial to slow down disease progression. Serum analysis may show nonspecific changes such as normal uric acid levels during an acute flare where 12%-43% of patients have normal to low levels [12]. This so-called normal uric acid in the acute setting may be related to the acute phase where uric acid tends to be reduced due to the up-regulation of interleukins and NLRP3 inflammasome. The NLRP3 inflammasome complex is a type of pattern-recognition receptor which is known to recognize MSU crystals. It is a known regulator of the innate inflammatory response in gout where studies have demonstrated that it is critical in sensing MSU deposition [15]. Definitive diagnosis requires joint aspiration and visualization of negative birefringent urate crystals under polarized light. Imaging also plays a role in diagnosing the disease whereas plain radiographs may show erosive changes which are from longstanding inflammation. Magnetic resonance imaging (MRI) is often helpful in identifying tophi. Ultrasonography allows for the identification of joint abnormalities and is highly sensitive and specific for urate crystal depositions. Dual-energy computed tomography (DECT) is a non‐invasive method for the visualization, characterization, and quantification of MSU crystal deposits based on their relative absorption of X-rays at different photon energy levels. This method has been employed to distinguish between gout and pseudogout (calcium crystals) [13].

Treatment of acute gout flares aims to decrease inflammation and includes corticosteroids, NSAIDs, and colchicine. Long-term treatment of gout consists of urate-lowering agents that promote the dissolution of MSU crystals. The American College of Rheumatology recommends treatment with allopurinol, a xanthine oxidase inhibitor, as a first-line option for patients. Another option in this class of medications that can be used is febuxostat. The goal is to reduce serum urate levels to 6 mg/dL or less, or less than 5 mg/dL in patients with extensive tophi deposits, by increasing the dosage every two to five weeks. Unlike allopurinol, which decreases the formation of uric acid, pegloticase is another agent that can be effective by converting uric acid into a more soluble compound that is easier to excrete in the urine. Although pegloticase is not recommended as first-line due to its high cost, it can be considered in patients with tophi or flare-ups despite being on maximal therapy [16]. Early treatment of acute gout flares and subsequent long-term therapy to reduce uric acid levels has been shown to significantly reduce disease progression to tophaceous gout and chronic gouty arthritis [13].

## Case presentation

**Figure 1:**
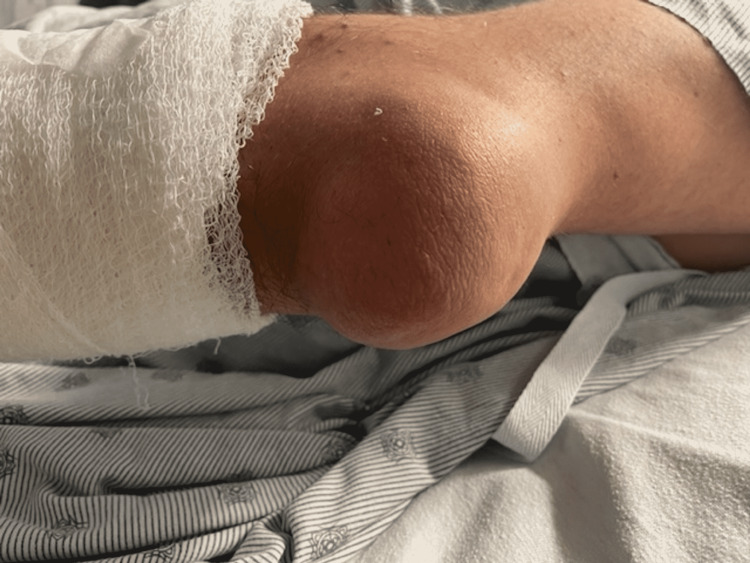
Large tophi on left elbow

**Figure 2:**
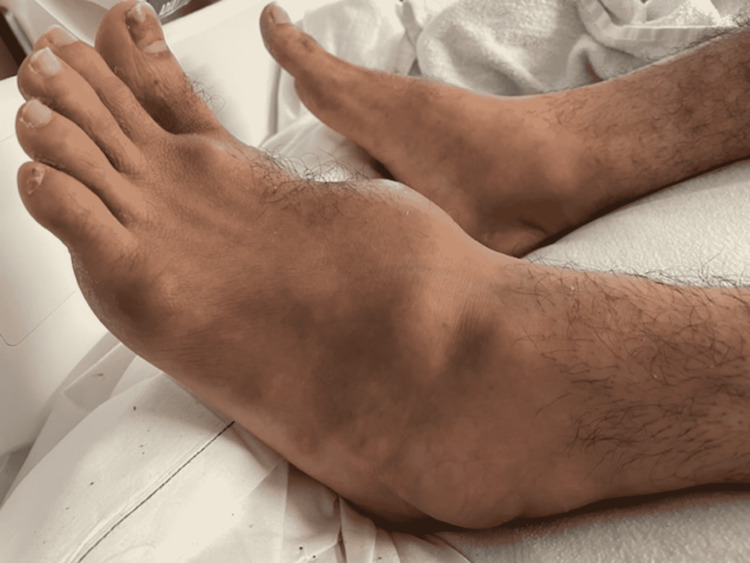
Multiple fluctuant lesions on bilateral feet mimicking abscesses

We present a 33-year-old Guatemalan male with a history of alcoholism who presented to the emergency department with multiple swellings on his extremities. The patient complained of mild pain with swellings on several joints of his feet, knees, and hands that developed several months prior. The patient also had large nodules on his elbows which he stated had been present for three years and were associated with pain and low-grade fevers with daily alcohol use. The patient stated he works in landscaping and had a chronic history of daily alcohol use. The patient noted difficulty walking secondary to the swelling in his bilateral foot and ankle joints. The patient was not on any medications. The patient’s vital signs were within normal limits and his BMI was 29.1 kg/m2. Physical examination revealed multiple fluctuant lesions of the bilateral elbow joints, see Figure 1, and knee joints as well as on the hands and feet, see Figure 2. Lesions of the elbows and left foot were fluctuant, tender to palpation, and erythematous, similar to the appearance of abscesses, while the remainder were non-tender. Initial laboratory findings were significant for elevated inflammatory markers, including erythrocyte sedimentation rate (ESR) 105 (normal: 0-15 mm/hr), C-reactive protein (CRP) 17.69 (normal: <10 mg/L), lactic acid 3.6 (normal: 0.5-2.2 mmol/L), and white blood cells (WBC) 16.14x10^3 (normal: 4 x10^3 11x10^3 uL). Uric acid level was 6.0 (normal: 3.5 to 7.2 mg/dL). Creatinine level was 1.1 (normal: 0.8 to 1.2 mg/dL) and glomerular filtration rate (GFR) was within the normal range. Human leukocyte antigen (HLA) B27 was negative and the HLA B5801 level was not ordered. The patient remained afebrile and hemodynamically stable throughout hospitalization. Podiatry, infectious disease, and rheumatology services were all consulted on this case. Infectious disease workup, including blood and urine cultures as well as HIV, rapid plasma reagin (RPR), Lyme disease, hepatitis panel, chlamydia, and gonorrhea was all unremarkable which directed the management to discontinue antibiotics. MRI findings were consistent with tenosynovitis and soft-tissue edema without acute osseous abnormalities. Nuclear medicine bone scan and Indium-labeled WBC scan showed numerous foci of white blood cell uptake in the soft tissue suggesting an acute inflammatory process that prompted the addition of steroids to treatment. A lesion on the dorsal aspect of the first metatarsophalangeal joint of the left foot was aspirated and drained thick, chalky fluid consistent with tophaceous gout and the cytology revealed uric acid crystals with negative birefringence under polarized light as well as no bacterial growth after 72 hours.

Once the diagnosis of gout was made, the rheumatology consultant recommended continuing the IV steroids and starting colchicine. The patient’s symptoms began to improve with this therapy. The patient was discharged on colchicine, allopurinol, and prednisone with instructions for close outpatient follow-up.

## Discussion

Typical gout flares present as a monoarticular swelling with associated pain, erythema, and warmth [17-19]. Polyarticular gout is more commonly seen in chronic or untreated gout patients over 10 years and may present with tophi, which appear as firm nodules [18]. Our patient differs from a typical gout presentation in that he presented with multiple large fluctuant lesions resembling abscesses on multiple joints throughout the body before having a diagnosis of gout.

According to Kumar et al. [13], presenting initially with widespread tophi is rare and is called gout nodulosis, where only a few cases have been reported in the literature. Several nodular lesions have been described in the past which is what brought about the term "gout nodulosis" to be its own entity to describe tophi as a presenting manifestation of gout. Cases reviewed in the literature presenting with gout nodulosis do not describe the lesions as fluctuant, but as firm nodules, making our patient extremely unique. Additionally, tophaceous deposits generally develop after 10 years of untreated gouty arthritis, where our patient stated he first developed the nodules three years prior [20]. 

It is mentioned that gout can be misdiagnosed especially in the younger population, as gout has a propensity to target older adults. This delays treatment leading to aggravation of the disease process affecting patients’ quality of life [18,19]. Gout was not part of the original differential diagnosis as our patients’ lesions resembled abscesses, leading us toward an infectious etiology. Therefore, it is important to consider gout in patients presenting with polyarticular lesions even if they are fluctuant and do not have the typical tophaceous appearance. It is also imperative to obtain aspiration of the lesion for accurate diagnoses, such as in our patient.

Although the patient presented with an acute flare on bilateral foot and ankle joints, the chronic tophaceous-appearing lesions on bilateral elbows with the patient’s history of these lesions being present for multiple years made this an acute on chronic presentation. Based on the American College of Rheumatology (ACR), patients with one or more subcutaneous tophi, strongly recommend initiating urate-lowering therapy. Therefore, the rheumatology consultant started the patient on allopurinol in addition to the treatment for the acute flare.

## Conclusions

We present this case to highlight the importance of investigating polyarticular joint disease and to consider gouty arthritis in a patient with multiple soft tissue swellings. Polyarticular disease as an initial presentation should warrant immediate work-up for infectious, inflammatory, and autoimmune etiologies. Diffuse tophaceous gout, as seen in our patient, presented atypically such that several of his lesions were fluctuant and tender, resembling abscesses. However, his negative blood cultures and lack of improvement with antibiotics warranted a rheumatological workup. In addition, although his uric acid levels were within normal levels, he was still started on urate-lowering therapy per ACR guidelines due to his multiple tophi. It is important to keep the differential diagnosis broad to include diseases with both common and uncommon presentations. 
